# Presence of bacteria in failed anterior cruciate ligament reconstructions

**DOI:** 10.1186/s40064-015-1213-2

**Published:** 2015-08-28

**Authors:** N Luisa Hiller, Aakash Chauhan, Michael Palmer, Sameer Jain, Nicholas G Sotereanos, Gregory T Altman, Laura Nistico, Rachael Kreft, J Christopher Post, Patrick J Demeo

**Affiliations:** Department of Biological Sciences, Carnegie Mellon University, Pittsburgh, PA USA; Department of Orthopaedic Surgery, Allegheny General Hospital, Pittsburgh, PA USA; Surgical Operations Squadron, 88th Medical Group, Wright-Patterson Air Force Base, Dayton, OH USA; Center for Excellence in Biofilm Research, Allegheny Singer Institute, Allegheny General Hospital, Pittsburgh, PA USA; Department of Surgery, Allegheny General Hospital, Pittsburgh, PA USA; Drexel University College of Medicine, Philadelphia, PA USA; Temple School of Medicine, Philadelphia, PA USA

**Keywords:** Anterior cruciate ligament reconstructions, Bacterial composition, IBIS Universal Biosensor, Broad-range PCR and high performance mass spectrometry

## Abstract

**Background:**

Novel microbial detection technologies have revealed that chronic bacterial biofilms, which are recalcitrant to antibiotic treatment, are common in failed orthopedic procedures.

**Questions:**

Are bacteria present on failed anterior cruciate ligament (ACL) reconstructions? Is there a difference in the presence or nature of bacteria in failed ACL reconstructions relative to a control set of healthy ACL’s?

**Methods:**

We used a case–control study design, where we analyzed the bacterial composition of 10 failed ACL reconstructions and compared it to 10 native ACL’s harvested during total knee arthroplasty. The IBIS Universal Biosensor was used to determine the nature of bacteria on ACL specimens, and fluorescent in situ hybridization (FISH) was used to visualize bacteria in a subset of cases.

**Results:**

Bacteria are present in failed ACL reconstructions. Bacteria are present in ACL’s harvested during total knee arthroplasty, but the nature of the species differs significantly between experimental and control sets. Twelve genera were detected in the experimental set (in both allografts and autografts), and in four samples multiple species were detected. In contrast, the control group was characterized by presence of *Propionibacterium acnes*.

**Conclusions:**

We demonstrate the presence of bacteria on failed ACLs surgeries, and open the door to investigate whether and how bacteria and the associated immune responses could possibly contribute to graft failure.

**Clinical relevance:**

If microbial pathogens can be linked to failed grafts, it could provide: (1) markers for early diagnosis of abnormal healing in ACL surgeries, and (2) targets for early treatment to prevent additional reconstruction surgeries.

## Background

Revision anterior cruciate ligament (ACL) surgery is a challenging problem for orthopaedic surgeons and their patients as long-term outcomes of revision ACL surgery have been shown to be inferior to primary ACL reconstructions (Wright et al. 2012). Failed primary ACL reconstructions can be attributed to traumatic rupture of the graft, technical error, failure of the graft to incorporate, biologic factors, or a combination of the above (MARS Group et al. 2010). When using standard culture to detect infections, revision ACL surgery secondary to infection is reported in less than 1 % of all primary ACL reconstructions (Barker et al. 2010; Burks et al. 2003; Hettrich et al. 2013; Indelli et al. 2002; Katz et al. 2008; Matava et al. 1998; McAllister et al. 1999; Williams et al. 1997); diabetes is a major risk factor associated with post-operative infection (Brophy et al. 2015). When present, deep infection can hinder the ability of the graft to incorporate into the femoral or tibial tunnels, or weaken the structure of the fibers of the graft. A combination of mechanical and biologic factors can cause tunnel lysis or enlargement which leads to long-term instability of the graft (Wilson et al. 2004). Some studies did not observe a significant difference in the rate of infection between allografts and autografts, (Burks et al. 2003; Katz et al. 2008), while a large (>2000) patient cohort noted an association between lower rate of infection and autografts (Brophy et al. 2015).

The rate of infections in orthopedic infections has been severely underestimated since clinical infections are usually detected by microbial cultures (Costerton et al. 2011). Studies on culture-negative revision arthroplasties, osteomyelitis, and bone fractures often reveal bacterial DNA and/or direct visualization of bacteria by microscopy (Costerton et al. 2005; Floyed and Steele 2003; Palmer et al. 2014; Tunney et al. 1998) (Gallo et al. 2011; Stoodley et al. 2008). This discrepancy is a result of the differences in metabolism between non-adherent planktonic bacteria and the slime-enclosed communities (termed biofilms) characteristic of chronic infections, which are resistant to growth on agar medium. Bacteria growing in chronic biofilms often do not invade host tissues or release toxins, and consequently can remain undetected for months or years (Costerton et al. 2007). An infectious etiology behind failed ACL reconstruction that has not been evaluated specifically is the presence of bacteria on reconstructed ACL grafts and their potential long-term effects.

In this study we applied the Ibis Universal Biosensor, which integrates polymerase chain reactions (PCR) and mass spectroscopy (MS) to detect and characterize bacteria on failed ACL grafts and compared them to native ACL’s harvested during total knee arthroplasty. In a subset of cases the bacteria was visualized by confocal microscopy using fluorescent in situ hybridization (FISH) with probes targeted at species-specific regions of the 16S rRNA gene. The goal of this study was to test the hypothesis that bacteria are present on failed ACL grafts, and that the bacterial species differ between failed ACL grafts and native ACL’s harvested during total knee arthroplasty.

## Results

### Description of subjects and detection technology

The experimental group was composed of 4 males and 6 females with a mean age of 30 years old undergoing revision surgery for failed primary ACL reconstruction. The primary grafts were three allografts and seven autografts. The control group was composed of 6 males and 4 females with a mean age of 68 years old undergoing a primary total knee arthroplasty. The material analyzed consisted of tissue, aspirate and/or synovial fluid obtained intra-operatively. Arthroscopic evaluation during revision ACL reconstruction and direct clinical analysis during the total knee arthroplasties did not reveal signs of clinical infection at the time of surgery (Table 1).

**Table 1:** Health and demographics of subject set

Study group	Estimated inter surgery time	Age at time of study Sx	Gender	BMI	Race	ACL of interest	Symptomology	Findings	Clinical Impression	Failed graft type	Reason for failure
Experimental	8	33	M	28.0	Caucasian	Right	Swelling	Tenderness, swelling, drainage (blood), instability	No infection	Autograft	Mechanical
Experimental	11	38	F	29.1	Caucasian	Right	Pain, swelling, stiffness, instability	Swelling, pain on motion	No infection	Hamstring autograft and BTB patellar autograft	Trauma
Experimental	3	30	M	33.1	Caucasian	Right	Pain, instability	Instability	No infection	Patellar tendon autograft	Mechanical
Experimental	6	22	M	27.4	Caucasian	Left	Pain	Swelling	No infection	Patellar tendon autograft	Trauma
Experimental	8	17	F	20.1	Caucasian	Right	Pain	Decreased ROM, pain	No infection	Hamstring autograft	Trauma
Experimental	14	40	F	25.1	Caucasian	Right	Pain, instability	Pain	No infection	Allograft	Mechanical and technical
Experimental	7	46	F	31.8	Caucasian	Left	Pain, Swelling	Swelling, decreased ROM, pain on motion	No infection	Allograft	Trauma
Experimental	1	23	M	29.8	Caucasian	Left	Pain, Swelling	Swelling, Pain on motion	No infection	Hamstring autograft	Trauma
Experimental	7	25	F	20.3	Caucasian	Right	Pain, numbness, instability	Pain	No infection	Posterior tibialis allograft	Mechanical
Experimental	4	22	F	23.5	Caucasian	Right	Instability	None	No infection	Patellar tendon autograft	Trauma
Control	NA	72	M	46.2	Caucasian	Left	Pain	Pain on motion	No infection	NA	NA
Control	NA	72	F	22.1	Caucasian	Right	Pain	Decreased ROM, pain on motion	No infection	NA	NA
Control	NA	64	M	37.6	Caucasian	Left	Pain	Decreased ROM, pain on motion	No infection	NA	NA
Control	NA	61	F	30.3	Caucasian	Left	Pain	Decreased ROM, pain on motion	No infection	NA	NA
Control	NA	66	F	30.4	Caucasian	Left	Pain	Tenderness, decreased ROM	No infection	NA	NA
Control	NA	63	M	32.7	Caucasian	Right	Pain	Decreased ROM, pain on motion	No infection	NA	NA
Control	NA	74	M	29.4	Caucasian	Right	Pain	Tenderness, decreased ROM, pain with motion	No infection	NA	NA
Control	NA	75	F	30.9	Caucasian	Left	Pain	Swelling, tenderness, decreased ROM, pain on motion	No infection	NA	NA
Control	NA	52	M	31.5	Caucasian	Left	Pain	Swelling, tenderness, decreased ROM, pain on motion	No infection	NA	NA
Control	NA	70	M	38.9	African Am.	Left	Pain	Tenderness, decreased ROM, pain with motion	No infection	NA	NA

*NA* not applicable, *ROM* range of motion, *BTB* bone-patellar tendon-bone.

Microbial detection was performed on the PCR-MS platform using the BAC detection plate. This analysis does not require prior knowledge of bacterial presence and is independent of traditional culture. The PCR primers are targeted at the universally conserved 16s rRNA gene and are optimized to classify the most abundant species within the samples. The BAC detection plate is extremely sensitive to Staphylococci since it contains additional primers targeting at this genus. Furthermore, primers targeted at the 23S rRNA gene will capture *Candida* sp and *Saccharomyces* sp, which were not detected in this study suggesting the ACLs were not infected with these fungal species.

### Microbial composition of ACLs

**Fig. 1:**
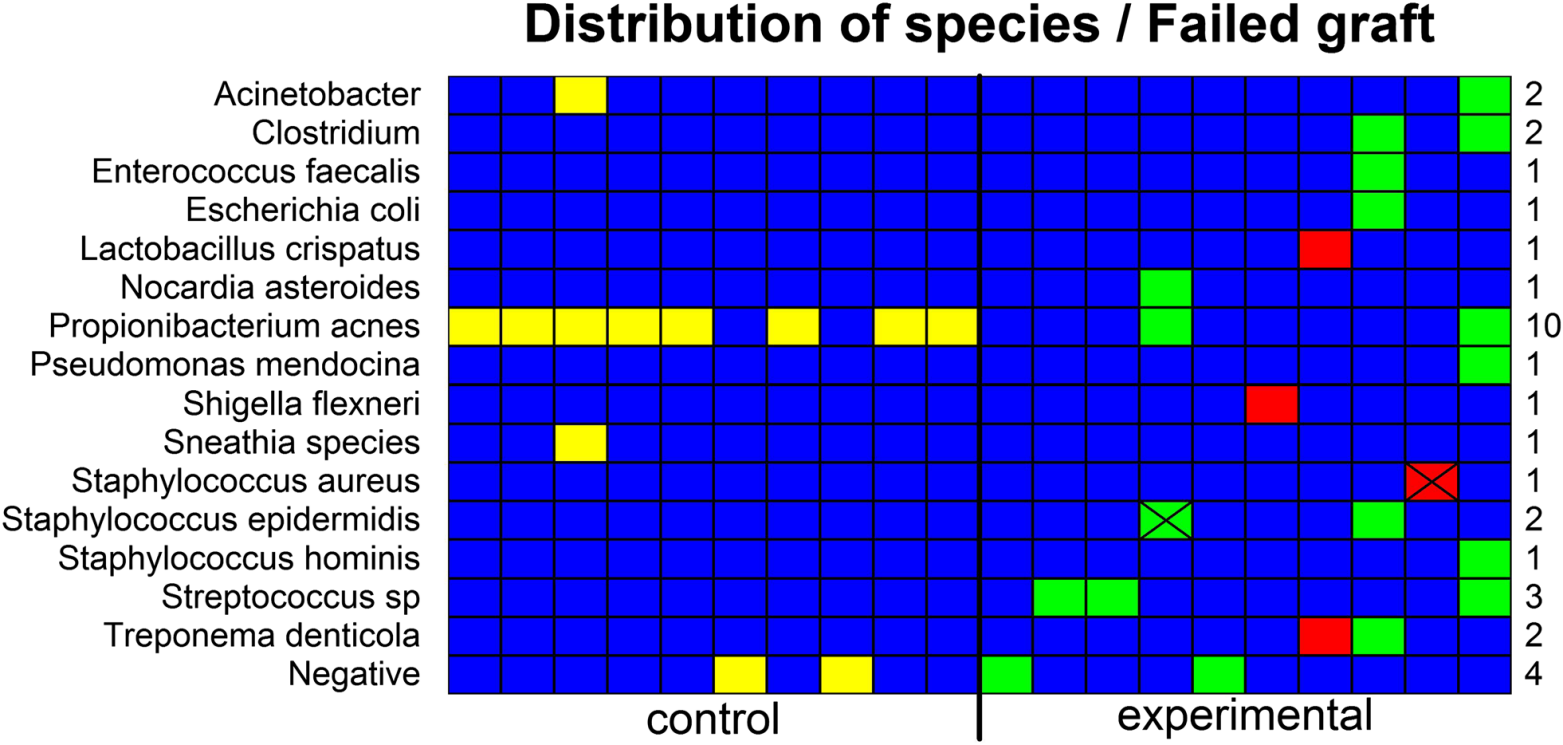
Bacterial composition of ACL samples. *Left side* control set of ACLs from knee arthroplasties. *Right side* experimental set of ACLs from failed grafts including autografts (*green*) and allografts (*red*). ‘X’ marks gene for methicillin resistance.

The PCR-MS identified bacteria in both the experimental and control samples, but the nature of the bacteria detected differed significantly between the sets (Fig. 1). Eighty percent of ACLs from failed grafts (including both the autografts and allografts) demonstrated evidence of bacterial DNA: *Staphylococci* (4/10), *Streptococci* (3/10), *Clostridium* (2/10), *Propionibacterium acnes* (2/10) and Treponema denticola (2/10), as well as single cases of *Acinetobacter, Enterococcus faecalis, Escherichia coli, Lactobacillus crispatus, Nocardia asteroides, Pseudomonas mendocina,* and *Shigella flexneri.* In contrast, the control group was characterized by presence of *Propionibacterium acnes* (8/10). Four experimental patients showed evidence of multiple species: one patient with *N. asteroides*, *P. acne,* and *S. epidermitis;* a second with *Lactobacillus crispatus* and *T. denticola;* a third with *Clostridium, Enterococcus faecalis, Escherichia coli, T. denticola* and *S. epidermitis*; and a fourth with *Acinetobacter, Clostrium, Propibacteirum acnes, Pseudomonas mendocina, Staphylococcus hominis,* and *Streptococcus sp* (Fig. 1). Eighty percent of the control group demonstrated evidence of bacterial DNA, but in this set the defining species was *Propionibacterium acnes* (8/10). In one control patient *Acinetobacter* and *Sneathia* were also detected. The gene coding for methicillin resistance was encountered in two patients from the revision ACL group.

**Fig. 2:**
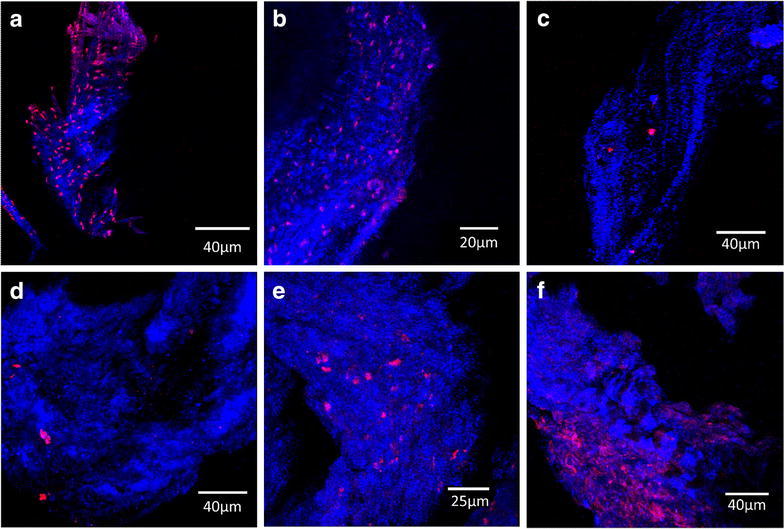
Confocal images of ACLs visualized using fluorescent in situ hybridization (FISH) targeted at 16S ribosomal RNA from *Staphylococcus sp* (**a**), *Streptococcus* (**b**, **d**), *Lactobacillus sp* (**c**), and *P. acnes* (**e**) on four experimental samples (one is co-infected with *Streptococcus sp. and P. acnes)*, and *P. acnes* on a control sample (**f**). *Red* corresponds to the bacteria and *blue* to reflected light from the tissue.

In four experimental samples and one control sample, where sufficient tissue was available, a secondary method of analysis was applied. These samples were stained by FISH and visualized by confocal microscopy (Fig. 2). Probes were selected based on the species detected by the PCR-MS analysis specifically: *Staphylococcus sp, Streptococcus sp.,Lactobacillus* sp and *P. acnes*. These probes bind 16S rRNA, thus the stained bacteria was metabolically active at the time of fixation and washing. We do not demonstrate a causal link between bacterial presence and ACL failure. However, the presence of bacteria in these failed ACL supports further investigations to determine whether there is a role for bacteria in tissue degeneration and/or host inflammation.

## Discussion

The overall ACL revision rate varies in the literature, with the most recent longitudinal long-term results from the MOON group reporting an overall 7.7 % revision rate of the ACL reconstructed knee at 6 years follow-up (Hettrich et al. 2013). Management of septic arthritis following ACL reconstruction requires immediate arthroscopic irrigation and debridement. The rate of removal and replantation of the graft varies in the literature, with recent evidence to suggest that immediate irrigation and debridement with retention of the graft may lead to acceptable results at 5 years of follow-up (Windhamre et al. 2014; Burks et al. 2003; Maletis et al. 2013; McAllister et al. 1999; Schulz et al. 2007; Williams et al. 1997).

Currently infections are monitored using standard culture techniques, and are reported in less than 1 % of ACL reconstructions (Barker et al. 2010; Burks et al. 2003; Hettrich et al. 2013; Indelli et al. 2002; Katz et al. 2008; Matava et al. 1998; McAllister et al. 1999; Williams et al. 1997). Our PCR-MS analysis detected bacteria in eighty percent of the experimental samples, suggesting that bacterial presence is ACLs is significantly underestimated by current techniques. This is consistent with studies on other types of orthopedic infections where bacteria were present in culture-negative orthopedic infections (Costerton et al. 2011; Jacovides et al. 2012; Palmer et al. 2014; Stoodley et al. 2011a, b; Mariscalco et al. 2014).

The most common culture positive pathogens associated with septic arthritis after ACL reconstruction are *S. epidermis* and *S. aureus* (Barker et al. 2010; Burks et al. 2003; Hettrich et al. 2013; Indelli et al. 2002; Katz et al. 2008; Matava et al. 1998; McAllister et al. 1999; Williams et al. 1997; Windhamre et al. 2014; Maletis et al. 2013). We detected these species in 3/10 of the experimental set and none of the control set. The bacterial species identified in the experimental sample are common human flora, and have been associated with both commensal and pathogenic states. Multiple genera identified in the experimental set have been identified in other types of orthopedic infections including Staphylococci, Streptococci, and Treponema. These three genera are oral colonizers, thus it is possible that these oral bacteria gain access to the circulation and establish a local infection when they encounter damaged tissue. The route of entry and its clinical significance remain open to debate and should be addressed in future studies.

While bacteria were detected in both experimental and control groups, the species composition varied between these groups. Multiple species of known human pathogens were encountered in the failed grafts. In contrast, arthroplasty samples were characterized by the presence of *P. acnes—*a common skin commensal. These results are consistent with the hypothesis that the activation of either bacterial regulatory pathways and/or host inflammatory responses affects the healing of ligaments and bone that are required in the repair of ACL damage. Alternatively, it is possible that these bacteria are present but do not play a role in graft failure. The present of *P. acnes* in the control samples from knee arthoplasties underscores the importance of characterizing the specific species and suggests that presence of bacteria alone does not implicate ACL damage. In fact, it is possible that under some conditions certain bacterial species could be beneficial by competing away pathogens or modulating inflammation. The correlation between individual species, host response, and damage is an import next step.

Failed ACL reconstructions can be attributed to many causes and our study shows that bacteria are commonly present within reconstructed ACL grafts irrespective of the type of graft used. A chronic, indolent bacterial infection could contribute to a significant weakening of the graft and create a chronic inflammatory environment, which could cause further compromise to the grafts integrity. This hypothesis will require a future study to analyze the cause and effect of chronic bacterial infections on the structural integrity of the ACL graft over time. Our belief is that bacterial presence contributes to failure of ACL reconstructions, which are likely the result of many contributing factors (e.g. mechanic and biologic). If subclinical chronic bacterial infections play a role in ACL graft failure, it will open the door for the development of diagnostics and treatments, targeted at the bacteria or host inflammatory response, to prevent ACL graft failure in the future.

## Methods

### Study design and sample preparation

Subjects for this study were recruited at the Department of Orthopaedic Surgery at Allegheny General Hospital, and approved by the Institutional Review Board (approval number FWA00015120). A total of twenty ACLs were collected from 20 patients. The experimental group consisted of ten ACL’s from ten patients undergoing revision surgery for failed primary ACL reconstructions. The control group consisted of ten native ACL’s from ten patients undergoing total knee arthroplasty without any prior surgery. The average time to revision surgery was 7 years.

Specimens were collected under sterile conditions in the operating room, placed immediately into a RNA stabilization agent (RNAlater, Qiagen, Germantown, MD) and stored at −80 °C for evaluation with the IBIS T5000 Universal Biosensor System and/or FISH analysis.

### DNA extraction and IBIS universal biosensor bacteria, antibiotic resistance, and *Candida* (BAC) detection assay for microbial identification

For DNA extraction the ACL was placed into a sterile microcentrifuge tube containing ATL Lysis buffer (Qiagen, Germantown, MD, cat# 19076) and proteinase K (Qiagen, cat# 19131). Samples were incubated at 56 °C until lysis. 100 μl of a mixture containing 50 μl each of 0.1 and 0.7 mm Zirconia beads (Biospec cat# 11079101z, 11079107zx respectively) were added to the samples which were then homogenized for 10 min at 25 Hz using a Qiagen Tissuelyser. Nucleic acid from the lysed sample was then extracted using the Qiagen DNeasy Tissue kit (Qiagen cat# 69506).

For microbial detection, 10 μl of each sample was loaded per well onto the BAC detection PCR plate (Abbott Molecular, cat# PN 05N13-01). The BAC detection plate is a 96 well plate which contains 16 primers that survey all bacterial organisms by using the omnipresent loci (e.g. 16S rRNA gene sequence), while some are targeted to specific pathogens of interest (e.g. the Staphylococcus-specific *tuf*B gene). The plate also includes primers for the detection of *Candida* species and some antibiotic resistance markers (e.g. mecA, vanA, vanB, and KPC). An internal calibrant of synthetic nucleic acid template is also included in each assay, controlling for false negatives (e.g. from PCR inhibitors) and enabling a semi-quantitative analysis of the amount of template DNA present. PCR amplification was carried out and the products were desalted in a 96-well plate format and sequentially electrosprayed into a mass spectrometer. The spectral signals were processed to determine the identities of the pathogens and a semi-quantitative determination of their relative concentrations on the ACLs (Ecker et al. 2008).

### Fluorescent in situ hybridization (FISH)

Aliquots of the ACL samples were fixed with fresh 4 % paraformaldehyde and incubate for 2–4 h at 4 °C. After the incubation the specimen was spun down and the supernatant removed, this process was repeated twice with Hank’s Salt Saline Solution (HBSS). Next, the samples were resuspended in 50 % Ethanol-PBS solution and stored at −20 °C for evaluation with FISH. FISH was performed as described by Nistico et al. (Nistico et al. 2014), using species-specific and genus-specific fluorescent 16 s rRNA probes. The bacteria targeted and probe sequences selected were: (1) *Streptococcus* “GTG ATG CAA GTG CAC CTT” (Kempf et al. 2000); (2) *Staphylococcus sp “*TCC TCC ATA TCT CTG CGC” (Trebesius et al. 2000); and (3) *Lactobacillus* sp “CCATTGTGGAAGATTCCCT” (Quevedo et al. 2011).

Samples were observed with Confocal Scanning Laser Microscopy (CSLM) imaging using a Leica DM RXE microscope attached to a TCS SP2 AOBS confocal system (Leica Microsystems, Exton, PA) using a 63X (NA1.2) water immersion lens.
